# Fumigation and contact activities of 18 plant essential oils on *Villosiclava virens*, the pathogenic fungus of rice false smut

**DOI:** 10.1038/s41598-019-43433-x

**Published:** 2019-05-14

**Authors:** Jingge Zheng, Tingting Liu, Zhixin Guo, Lan Zhang, Liangang Mao, Yanning Zhang, Hongyun Jiang

**Affiliations:** Key Laboratory of Control of Biological Hazard Factors (Plant Origin) for Agri-product Quality and Safety, Ministry of Agriculture and Rural Affairs of the People’s Republic of China, Institute of Plant protection, Chinese Academy of Agricultural Sciences, Beijing, 100193 China

**Keywords:** Chemical biology, Natural products

## Abstract

Rice false smut (RFS), caused by *Villosiclava virens*, is an emerging devastating disease of rice panicles worldwide and produces yield loss and mycotoxin residues in rice. In this study, 18 plant essential oils (PEOs) were selected to evaluate antifungal activity via fumigation and contact methods against the mycelial growth and conidial germination of *V*. *virens*. The primary compositions of PEOs with stronger fungistatic activity were analyzed using gas chromatography (GC)-mass spectrometry (MS), and the changes in the mycelial morphology were observed using scanning electron microscopy (SEM). Antifungal tests showed that cinnamon bark oil and cinnamon oil had stronger fumigation and contact effects on *V*. *virens* than the other oils tested. The primary active composition in both cinnamon bark oil and cinnamon oil was *trans*-cinnamaldehyde, which exhibited contact activities with EC_50_ values of 2.13 and 35.9 μg/mL against mycelial growth and conidial germination, respectively. The hyphae surface morphological alterations caused by cinnamon bark oil, cinnamon oil and *trans*-cinnamaldehyde included shriveling, vacuolation and exfoliation. In conclusion, cinnamon bark oil and cinnamon oil have the potential to prevent and control RFS, and *trans*-cinnamaldehyde is a promising natural lead compound for new fungicide discoveries to control RFS contamination and mycotoxin residues in rice.

## Introduction

Rice false smut (RFS) caused by *Villosiclava virens* is becoming one of the most economically important grain diseases in China with its incidence growing to 76% due to the expanded planting of hybrid rice cultivars, overuse of nitrogenous fertilizers and an apparent change in climate^1–3^. RFS can reduce the yield and quality of rice by converting the grains to false smut balls^4,5^ and produce a variety of mycotoxins, including ustiloxins and ustilaginoidins, which may be deleterious to human and animal health. Ustiloxins inhibit cell microtubule assembly and skeletal formation^6,7^. In addition, Institute of Cancer Research (ICR) mice showed a variety of lesions of the liver, heart and kidney after eating rice grains and feed contaminated with RFS balls^8^. Ustilaginoidins are bis-naphtho-γ-pyrone mycotoxins, some of which exhibit teratogenicity against ICR mouse embryo limb buds and midbrain cells and inhibitory activities against adenosine triphosphate synthesis in mitochondria^9,10^.

Chemical fungicides are currently a major approach to control RFS in China. Several fungicides, including demethylation inhibitors (DMI), such as tebuconazole, prochloraz, and propiconazole, are commonly applied^4,11^. However, the overuse and high frequency use of chemical pesticides could induce resistance, residue and environmental pollution risk^12^. Thus, the potential alternative of plant essential oils (PEOs) rather than chemical fungicides has been receiving increasing amounts of attention with the goal of developing green fungicides and techniques to control RFS.

Currently, many types of PEOs with potent and broad-spectrum antifungal activities are considered to be the candidates to guard agri-foods, herbal medicines and wood^13–15^. Previous studies demonstrated that peppermint oil showed strong antifungal activity on seven genera of fungi^16–18^ and thyme oil had inhibitory effect on fruit rot, toxigenic and wood-rot fungi, including *Colletotrichum*, *Fusarium*, *Phytophthora*, *Botryosphaeria*, *Aspergillus*, *Penicillium*, *Trametes* and *Laetiporus*^15,19,20^. Many small molecular compounds are the natural primary constituents of PEOs, account for their antifungal activity and have been registered as botanical fungicides, such as carvacrol, allicin, and eugenol^14,21^.

Some plant extracts have fungitoxic effects on *V*. *virens*. Awuah reported that steam distillate from the leaves of *Cymbopogon citratus* completely inhibited the mycelial growth of *V*. *virens*, followed by a hot water extract from the fresh leaves of *Ocimum gratissimum* with a reduction in mycelial growth of 60%, and hot water extracts from the dry fruits of *Monodera myrstica*, *Xylopia aethiopica* and *Chromoleana odorata* reduced radial growth by 1.9–12.6%^22^. Jin *et al*. demonstrated that 30% ethanol extracts of *Mentha arvensis* at a 10% concentration completely inhibited the germination of chlamdospores and conidiospores, and the inhibition by *Euphorbia helioswpia* was 14.79% and 7.23, respectively^23^. However, there is limited information about the control of RFS by PEOs.

Fumigation and contact activity assays are important choices to comprehensively evaluate the antifungal activity of PEOs^16,24^. PEOs are complex natural mixtures, which contain volatile components and other chemicals at different contents^18^. The fumigation activity assay is useful to test the antifungal activity of volatile chemicals, and a contact activity experiment can assess the other components with the exception of comparing the antifungal activity with the general fungicides^16,25^. Through scanning electron microscopic (SEM) observation, the PEOs resulted in the formation of vacuolation, shriveling, collapse and fracture in the pathogenic fungus in both fumigation and contact activity experiments^26,27^.

Thus, the specific contents of this study are (1) to evaluate the antifungal activity of 18 PEOs using fumigation and contact methods (2) to explore the primary active components of the PEOs with higher fungistatic activity using GC-MS analysis, and (3) to observe the morphological alteration of *V*. *virens* hyphae after treatment with PEOs with higher antifungal activity and their primary compounds using SEM.

## Results

### Antifungal evaluation of 18 PEOs *in vitro*

#### Antifungal effect of 18 PEOs on mycelial growth

The fumigation and contact effects of 18 PEOs against the mycelial growth of *V*. *virens* are shown in Table 1. In the fumigation activity assay, all the 18 PEOs showed inhibitory effects on the mycelial growth of *V*. *virens* at the concentration of 10 µL/L air *Acorus tatarinowii* rhizome oil, *Angelia dahurica* oil, cinnamon bark oil, cinnamon oil, *Litsea cubeba* oil, myrrh oil and thyme oil inhibited the mycelial growth completely, followed by peppermint oil, clove oil, holly oil and *Forsythia suspansa* oil that inhibited 74.1%, 72.1%, 67.7% and 60.4%, respectively. In contrast, *Artemisia argyi* oil, camphor oil, *Ligusticum wallichii* oil, myristica oil, *Platycladus orientalis* oil and tea seed oil showed less than 50% inhibition against mycelial growth of *V*. *virens*.

**Table 1:** Antifungal evaluation of 18 PEOs on the mycelial growth and conidial germination of *V. virens* using two methods.

Plant essential oil	Inhibition of mycelial growth (%)		Inhibition of conidial germination (%)	
	10 μL/L air Fumigation	20 μg/mL Contact	7.5 μL/L air Fumigation	40 μg/mL Contact
*Acorus tatarinowii* rhizome oil	100 ± 0.00a	3.60 ± 2.41b	100 ± 0.00a	0.00 ± 0.00b
*Angelica dahurica* oil	100 ± 0.00a	24.5 ± 4.12b	100 ± 0.00a	0.00 ± 0.00b
*Artemisia argyi* oil	21.2 ± 2.58 cd	5.74 ± 2.45b	0.00 ± 0.00c	0.00 ± 0.00b
Camphor oil	24.9 ± 2.77 cd	13.2 ± 2.27b	0.00 ± 0.00c	0.00 ± 0.00b
Cinnamon bark oil	100 ± 0.00a	66.1 ± 7.94a	100 ± 0.0a	100.00 ± 0.00a
Cinnamon oil	100 ± 0.00a	71.9 ± 0.875a	100 ± 0.00a	100.00 ± 0.00a
Clove oil	72.1 ± 14.0ab	12.4 ± 0.963b	0.00 ± 0.00c	0.00 ± 0.00b
*Dalbergia* wood oil	45.8 ± 2.21bc	26.9 ± 2.48b	0.00 ± 0.00c	0.00 ± 0.00b
*Forsythia suspansa* oil	60.4 ± 1.96b	13.9 ± 8.34b	0.00 ± 0.00c	0.00 ± 0.00b
Holly oil	67.7 ± 1.87b	13.4 ± 1.57b	0.00 ± 0.00c	0.00 ± 0.00b
*Ligusticum wallichi*i oil	46.6 ± 1.84bc	32.6 ± 1.35b	0.00 ± 0.00c	0.00 ± 0.00b
*Litsea cubeba* oil	100 ± 0.00a	14.5 ± 7.17b	100 ± 0.00a	0.00 ± 0.00b
Myristica oil	13.1 ± 3.30d	9.74 ± 1.69b	0.00 ± 0.00c	0.00 ± 0.00b
Myrrh oil	100 ± 0.00a	17.0 ± 2.12b	100 ± 0.00a	0.00 ± 0.00b
Peppermint oil	74.1 ± 3.81ab	14.5 ± 2.85b	0.00 ± 0.00c	0.00 ± 0.00b
*Platycladus orientalis* oil	12.2 ± 2.08d	16.4 ± 0.808b	0.00 ± 0.00c	0.00 ± 0.00b
Tea seed oil	4.37 ± 0.833d	10.5 ± 0.851b	0.00 ± 0.00c	0.00 ± 0.00b
Thyme oil	100 ± 0.00a	19.8 ± 0.450b	86.5 ± 1.01b	0.00 ± 0.00b

Data presented in table are mean ± SE. ^a–d^Significant differences at P < 0.05 level according to Scheffe’s multiple range test.

Alternatively, the inhibition of mycelial growth was 66.1% and 71.9%, respectively, after cinnamon bark oil and cinnamon oil treatment in the contact activity assay. *L*. *wallichii* oil, dalbergia wood oil and *A*. *dahurica* oil reduced only 32.6%, 26.9% and 24.5% of the mycelial growth, respectively. The other 13 PEOs showed lower than 20% inhibition.

According to the results of the antifungal evaluation of the 18 PEOs, the EC_50_ values of the PEOs with higher antifungal activity were estimated using probit analyses. Table 2 showed that both cinnamon bark and cinnamon oils exhibited the strongest antifungal activity by fumigation and contact methods with EC_50_ values less than 0.5 µL/L air and 4.28 and 4.47 µg/mL, respectively. Myrrh oil and *L*. *cubeba* oil showed strong fumigation activity with the EC_50_ values less than 0.5 µL/L air but no obvious contact activity. In contrast, dalbergia wood oil and *L*. *wallichii* oil exhibited moderate contact activity with EC_50_ values of 24.7 and 35.4 µg/mL, respectively, but not good fumigation activity. *A*. *dahurica* oil also showed fumigation activity with an EC_50_ value of 4.17 µL/L air, and thyme oil exhibited fumigation and contact activity with EC_50_ values of 20.8 µL/L air and 78.7 µg/mL, respectively.

**Table 2:** EC_50_ value of ten substances tested on.

Plant essential oils		Fumigation		Contact	
		Mycelial growth	Germination	Mycelial growth	Germination
*Acorus tatarinowii* rhizome oil	EC_50_	<0.5	<0.5	—	78.6
	**χ** ^**2**^	—	—	—	2.50
*Angelica dahurica* oil	EC_50_	4.17	1.46	—	78.9
	**χ** ^**2**^	2.39	1.28	—	7.52
Cinnamon bark oil	EC_50_	<0.5	<0.5	4.28	33.1
	**χ** ^**2**^	—	—	2.19	9.47
Cinnamon oil	EC_50_	<0.5	<0.5	4.47	30.9
	**χ** ^**2**^	—	—	2.99	9.37
*Dalbergia* wood oil	EC_50_	—	—	24.7	—
	**χ** ^**2**^	—	—	0.629	—
*Ligusticum wallichi*i oil	EC_50_	—	—	35.4	234
	**χ** ^**2**^	—	—	0.215	15.5
*Litsea cubeba* oil	EC_50_	<0.5	<0.5	—	—
	**χ** ^**2**^	—	—	—	—
Myrrh oil	EC_50_	<0.5	<0.5	—	64.2
	**χ** ^**2**^	—	—	—	3.81
Thyme oil	EC_50_	20.8	6.96	78.7	—
	**χ** ^**2**^	0.220	6.78	12.8	—
*Trans*-cinnamaldehyde	EC_50_	<0.5	<0.5	2.13	35.9
	**χ** ^**2**^	—	—	4.19	12.4

*V. Virens* under two conditions. Pearson χ^2^ statistic with P values indicating the goodness-of-fit for data to the expected probit response mode. “—” indicates that this essential oil has such low or high activity that the EC_50_ value cannot calculated or χ^2^ cannot be calculated with this method.

#### Inhibitory effects of 18 PEOs on conidial germination

Effects of the fumigation and contact of the 18 PEOs on the conidial germination of *V*. *virens* are shown in Table 1. In the fumigation activity test, *A*. *dahurica* oil, *A*. *tatarinowii* rhizome oil, cinnamon bark oil, cinnamon oil *L*. *cubeba* oil and myrrh oil exhibited 100% inhibition on conidial germination, and thyme oil inhibited 86.5% of the conidial germination of *V*. *virens* at the concentration of 7.5 μL/L air. In the contact activity tests, only cinnamon bark and cinnamon oils completely inhibited conidial germination. However, the other 16 PEOs had no significant inhibitory effect on the conidial germination of *V*. *virens*.

The results of the EC_50_ values of the PEOs with strong antifungal activity against the conidial germination of *V*. *virens* are shown in Table 2. Cinnamon bark and cinnamon oils showed the strongest inhibitory effect on the conidial germination in both the fumigation and contact activity assays with EC_50_ values less than 0.5 µL/L air and 33.1 and 30.9 µg/mL, respectively. *L*. *cubeba* oil exhibited strong fumigant activity with EC_50_ values less than 0.5 µL/L air but not good contact activity. In contrast, *L*. *wallichii* oil had moderate contact activity with an EC_50_ value of 234 µg/mL but little fumigation activity.

### The major constituent of PEOs with higher antifungal activities and its contact activity against *V*. *virens*

#### Major constituent of PEOs with higher inhibitory activity

**Figure 1:**
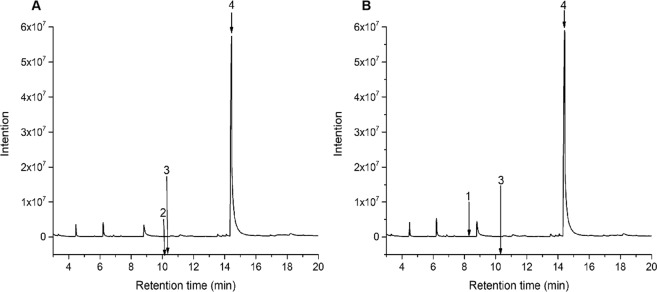
GC-MS chromatograms of the PEOs from (**A**) cinnamon oil and (**B**) cinnamon bark oil.

The major compounds of the cinnamon bark and cinnamon oils were analyzed using GC-MS and were listed in Table 3, along with their retention indices, compound names and relative contents. As presented in Fig. 1, the chemical components in cinnamon (Fig. 1A) and cinnamon bark (Fig. 1B) oils were satisfactorily separated under the chromatographic and MS conditions. The natural components identified from the cinnamon bark and cinnamon oils represented 71.47% and 71.46% of the total oils, respectively. The most abundant compound in both the cinnamon bark and cinnamon oils was *trans*-cinnamaldehyde. Its relative contents were 71.32% and 71.27%, respectively, in the two oils. These results were consistent with previous studies^15,28,29^. In addition, eucalyptol was found in the two oils, while o-cymene was identified solely in the cinnamon oil, and camphene was detected in the cinnamon bark oil.

**Table 3:** Natural major compositions of cinnamon bark oil and cinnamon oil.

Identified peaks	Retention index	Compound	Relative content (%)	
			Cinnamon oil	Cinnamon bark oil
1	943	Camphene	—	0.12
2	1042	o-Cymene	0.07	—
3	1059	Eucalyptol	0.07	0.08
4	1189	*Trans*-cinnamaldehyde	71.32	71.27
Total			71.46	71.47

#### Antifungal activity of *trans*-cinnamaldehyde on *V*. *virens*

The fumigation and contact activity of *trans*-cinnamaldehyde on *V*. *virens* was shown in Table 2. The EC_50_ values of *trans*-cinnamaldehyde against mycelial growth and conidial germination were both less than 0.5 µL/L air in the fumigation activity assay, and 2.13 µg/mL and 35.9 µg/mL in the contact activity assay, respectively.

### Mycelial morphology alteration of *V*. *virens*

**Figure 2:**
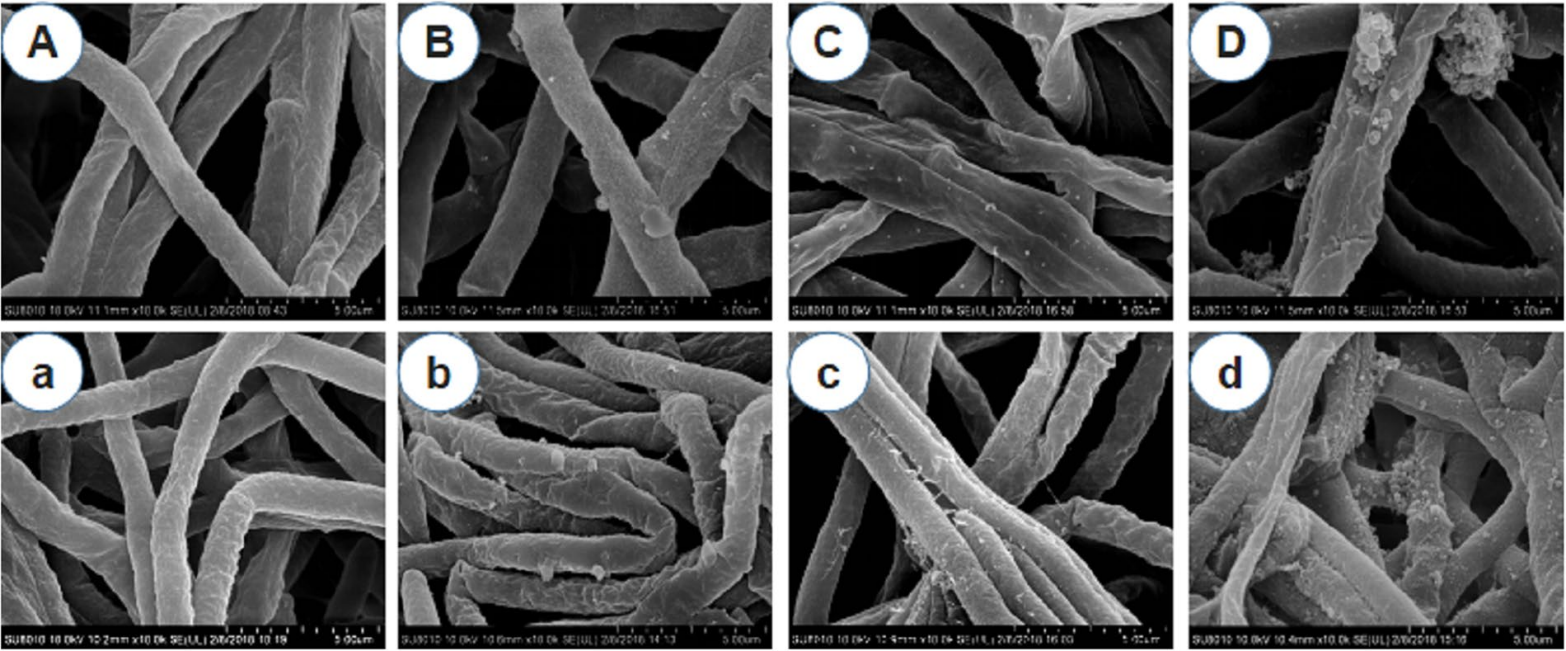
Scanning electron micrograph of *V. virens* hyphae. The pictures on the top are treated at 0.5 μL/L air in the fumigation activity assay. (**A**) Hyphae without PEOs or *trans*-cinnamaldehyde (control). (B–D) Hyphae treated with cinnamon bark oil, cinnamon oil and *trans*-cinnamaldehyde. On the bottom are treatments from the contact activity experiment at a concentration of 2 μg/mL. (a) Hyphae treated with acetone (CK). (b–d) Hyphae treated with cinnamon bark oil, cinnamon oil and *trans*-cinnamaldehyde. All of the magnifications are ×10,000.

SEM micrographs were used to determine the effect of cinnamon bark oil, cinnamon oil and *trans*-cinnamaldehyde on the surface morphology of the mycelia after seven days treatment at the concentrations of 0.5 μL/L air and 2 µg/mL by fumigation and contact activity methods, respectively. As shown in Fig. 2, the hyphae exposed to the chosen concentrations of the vapor of the PEOs or grown on toxic media showed degenerative changes in the hyphal morphology in comparison to the thick, elongated, smooth surfaced hyphae in the control plates (Fig. 2A,a). The abnormal phenomena caused by fumigation included: exfoliated flakes (Fig. 2B), applanate (Fig. 2C), shriveling (Fig. 2C,D) and vacuolation (Fig. 2D). Contact effect on the mycelial surface exhibited more serious exfoliated flakes damage (Fig. 2b), membrane injury (Fig. 2c), collapse and blistering (Fig. 2d).

From the aspects of the substances tested, the cinnamon bark oil induced the mycelia surface rough and ruptured, but cinnamon oil caused the mycelial shriveling, applanate and membrane injury. As shown in Fig. 2(D,d), *trans*-cinnamaldehyde aggravated the mycelial abnormalities of collapsing and blistering.

### Fungal sporulation difference of *V*. *virens*

Sporulation of *V*. *virens* was significantly affected by the tested substances (cinnamon bark oil, cinnamon oil and *trans*-cinnamaldehyde) under both fumigation and contact conditions. The results of fungal sporulation difference was shown in Table 4. Compared to the traditional contact method, fumigation method exhibited stronger inhibitory effect on the conidium sporulation. The fumigation treatment of *trans*-cinnamaldehyde completely inhibited the conidium sporulation of *V*. *virens*. Among the three tested substances, *trans*-cinnamaldehyde had the highest sporulation inhibition in the fumigation treatment method and cinnamon oil had the lowest inhibition under both treatment methods.

**Table 4:** Fungal sporulation difference of cinnamon oil, cinnamon bark oil and *trans*-cinnamaldehyde by two method at 35 µg/mL and 0.5 µL/L air, respectively.

Treatment	Fungal sporulation (×10^5^/mL)	
	Contact activity 35 µg/mL	Fumigation activity 0.5 µL/L air
Control	27.3 ± 4.33c	1033.3 ± 88.2d
Cinnamon oil	12.7 ± 1.19b	546.67 ± 24.0c
Cinnamon bark oil	3.49 ± 0.65a	280 ± 15.3b
*Trans*-cinnamaldehyde	5.73 ± 0.87ab	0.00 ± 0.00a

(Values are expressed as the means ± SE of three replicates, P < 0.05).

## Discussion

PEOs are one of the important sources of novel fungicides^30,31^. In this study, we evaluated the fumigation and contact activities of 18 PEOs against *V*. *viren*. Among the 18 PEOs, cinnamon bark and cinnamon oils showed the strongest fumigation and contact activities against the mycelial growth and conidial germination of *V*. *virens*. In the fumigation assay, 6 PEOs, including *A*. *dahurica* oil, *A*. *tatarinowii* rhizome oil, cinnamon bark oil, cinnamon oil, *L*. *cubeba* oil and myrrh oil completely inhibited the mycelial growth and conidial germination of this pathogen, and the other 12 PEOs had moderate inhibitory effects. In the contact activity test, cinnamon bark and cinnamon oils showed stronger antifungal activity on the mycelial growth and conidial germination than the other 16 PEOs. It is interesting that *L*. *cubeba* oil had stronger fumigation activity while *L*. *wallichii oil* showed stronger contact activity against mycelial growth and conidial germination of *V*. *virens*. Similar researches have been reported that volatile phase effects of oregano, rosemary and lavender essential oils were more effective on fungal growth of *P*. *infestans* and *B*. *cinerea* than contact phase effect^29,32^. However, some other PEOs showed the contrary antifungal activity by two methods. For example, thyme, clove and cinnamon proved to be better contact effect than fumigation activity against *P*. *roqueforti*, *P*. *corylophilum*, *Eurotium*. *repens* and *A*. *flavus*. The components of PEOs and species of fungi are the main influence factors on the inhibitory effect^33^. The results of this study indicated that cinnamon bark and cinnamon oils had the strongest antifungal effects on *V*. *virens*, with EC_50_ values of 4.28 μg/mL and 33.1 μg/mL, respectively, against mycelial growth and 4.47 μg/mL and 34.6 μg/mL, respectively, against conidial germination, which were lower than the values generally reported^14,32^. In previous studies, cinnamon bark oil exhibited strong contact toxicity against toxigenic and foodborne fungi, and cinnamon oil had contact inhibitory effect on wood-rot fungi^19,20,34,35^. Alternatively, since cinnamon bark and cinnamon oils are both from the *C*. *cassia* plant, which is commonly known as cinnamon, consumer acceptance is less of a problem because the US Food and Drug Administration (US FDA) lists cinnamon as a generally recognized as safe substance GRAS), and it is used in the fields of food, Chinese medicine, cosmetic and other materials around the world^17,35,36^. A comparison of these results revealed that cinnamon bark and cinnamon oils have the potential to prevent and control RFS.

To explore the possible active constituent of cinnamon bark oil and cinnamon oil, the major components of cinnamon bark oil and cinnamon oil were analyzed, and its contact activity against mycelial growth and conidial germination was evaluated. The photographs of cinnamon bark oil, cinnamon oil and *trans*-cinnamaldehyde with light microscope of *V*. *virens* hyphae can be seen in Supplementary Information in Figs S1 and S2. The results revealed that *trans*-cinnamaldehyde was the most abundant compound in both cinnamon bark and cinnamon oils and had a higher inhibitory effect on the mycelial growth than that of cinnamon bark and cinnamon oils. Similarly, several research papers reported that the fungicidal activity of cinnamon oil on wood-rot fungi and plant pathogenic fungi was attributed to its primary component, *trans*-cinnamaldehyde^14,19^. Many studies indicated that cinnmaldehyde could be used as an excellent candidate to control plant diseases caused by *Rhizoctonia*. *solani*, *F*. *oxysporum*, *A*. *flavus*, *C*. *gloeosporioides*, *A*. *citrii* and *B*. *cinerea*^14,20,37^. The results indicated that *trans*-cinnamaldehyde could be studied further to develop an eco-friendly and acceptable fungicide to protect rice from RFS and mycotoxin contamination.

The SEM micrographs indicated that the mycelia shrank and became applanate, exuvial and blistery after treatment with cinnamon bark oil and cinnamon oil. Soylu *et al*. found that *Origanum* essential oil caused the hyphal morphology of *B*. *cinerea* to degenerate, resulting in cytoplasmic coagulation, vacuolations, hyphal shriveling and protoplast leakage^16^, and Xu *et al*. also reported that *trans*-cinnamaldehyde induced the hyphae collapse and fracture of *A*. *alternata*^27^. As shown in the SEM graphs, cinnamon bark oil was more toxic than cinnamon oil, which might be related to the contents of camphene, eucalyptol, and o-cymene, since different chemical compounds stimulated or inhibited the mycelial growth alone or together^38^. The most serious disruption to the hyphae caused by *trans*-cinnamaldehyde indicated that it was the primary active compound in the cinnamon bark and cinnamon oils. Different abnormal phenomena might be responsible for the different treatments. As the previous study, fumigation impacts of PEOs on fungal structures might reflect effects of the volatiles emitted by oils on surface mycelial development and/or the perception/transduction of signals involved in the switch from vegetative to reproductive development^16^. The contact activity of PEOs may be impacted by interactions with the matrix components, which resulted in mycelial injury^39^. This result further confirmed that cinnamon bark oil, cinnamon oil and *trans*-cinnamaldehyde inhibited mycelial growth by destroying the surface structure of the mycelia and provided some reference and a basis to further explore the action mechanism of PEOs against *V*. *virens*. The specific mechanism on gene expressions and protein activities of cinnamon bark oil, cinnamon oil and *trans*-cinnamaldehyde against hyphae surface morphological alterations of *V*. *virens* remained unclear and needs to be studied.

**Figure 3:**
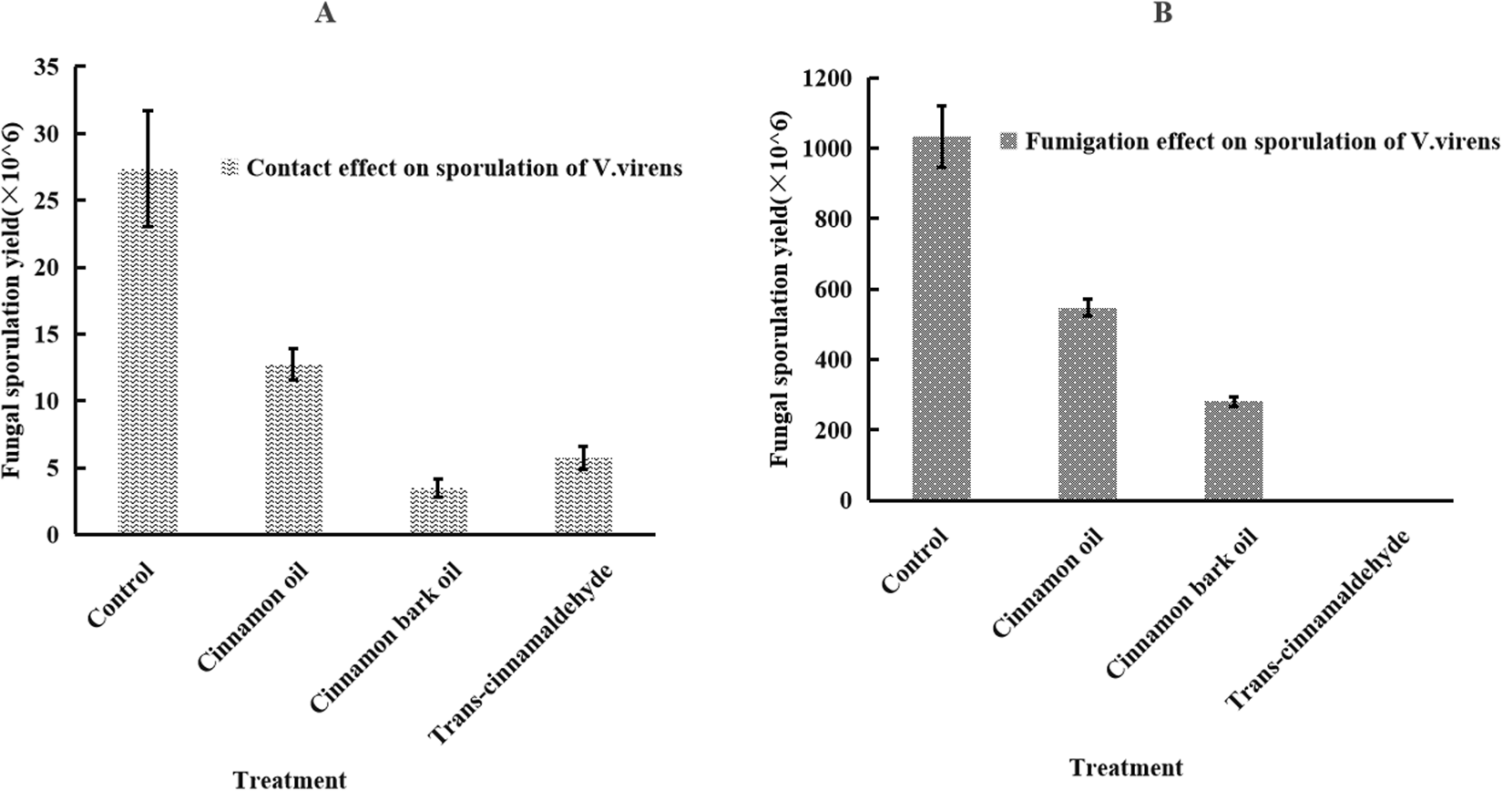
Histogram of three tested substances on fungal sporulation yield of *V. virens* by two methods. (**A**) Is the sporulation yield of *V*. *virens* treated with acetone (Control) and cinnamon oil, cinnamon bark oil and *trans*-cinnamaldehyde at the concentration of 35 µg/mL by contact method, (**B**) is the sporulation yields of *V*. *virens* treated without PEOs (Control) and with cinnamon oil, cinnamon bark oil and *trans*-cinnamaldehyde at the concentration of 0.5 µL/L air by fumigation method. Data are represented means ± SE of 3 replications.

To evaluate the inhibitory effect on reproductivity of *V*. *virens*. The sporulation difference of *V*. *virens* was conducted. As shown in the Fig. 3, cinnamon bark oil, cinnamon oil and *trans*-cinnamaldehyde reduced the fungal sporulation yields significantly compared with the normal sporulation yield (control). Among the three tested substances, cinnamon bark oil possessed strongest contact effect (Fig. 3B) and *trans*-cinnamaldehyde showed the strongest fumigation effect (Fig. 3A) on the sporulation of *V*. *virens*, which is not accordance with the antifungal effect of the tested substances against the conidial germination of *V*. *virens*. The reason for different antifungal effect on germination and sporulation haven’t been reported before and the different compounds in both PEOs and the interaction between chemicals and conidium might be the important factors and need further study.

In summary, cinnamon bark oil and cinnamon oil showed obvious fumigation and contact activity against the mycelial growth, conidial germination and sporulation of *V*. *virens*, and their primary active compound was *trans*-cinnamaldehyde. The SEM observations revealed that the hyphae were shriveled and exfoliated after *trans*-cinnamaldehyde treatment. The sporulation was significantly decreased after fumigation with *trans*-cinnamaldehyde. This study demonstrated that cinnamon bark and cinnamon oils possess the potential to be used as a bio-fungicide to prevent and control RFS, and *trans*-cinnamaldehyde is a very promising natural lead compound for the discovery of high efficacy and low risk fungicides. Up till now it has not been reported that any plant essential oils can be used to prevent and control RFS in the greenhouse or fields. It is an important topic to evaluate the efficay of cinnamon bark oil and cinnamon oil against this disease in fields in further studies.

## Materials and Methods

### Plant essential oils

In the study, thirteen PEOs were purchased from the Jiangxi Xinsen Natural Vegetable Oil Co. Ltd. (label a). Four PEOs and *trans*-cinnamaldehyde (98.3%) were obtained from the Jiangxi Cedar Natural Medicinal Oil Co. Ltd. (label b), and one PEO was purchased from the Jiangxi Hengcheng Natural Flavor Oil Co. Ltd. (label c). (Jiangxi, China). Detailed information about the PEOs is listed in Table 5. All the PEOs were stored in sealed vials at 4 °C for further analysis.

**Table 5:** Detailed information of 18 PEOs.

Local name	Plant Latin name	Plant part used	Family	Genus
*Acorus tatarinowii* rhizome oil^a^	*Acorus tatarinowii*	Root	Araceae	*Acorus* Linn.
*Angelica dahurica* oil^a^	*Angelica dahurica*	Stem, leaf and root	Umbelliferae	*Angelica* Linn
*Artemisia argyi* oil^a^	*Artemisia argyi*	Leaf	Compositae	*Artemisia* Linn.
Camphor oil^a^	*Cinnamonum camphora*	Trunk and branch	Lauraceae	*Cinnamomum* Trew
Cinnamon bark oil^a^	*Cinnamomum cassia*	Bark	Lauraceae	*Cinnamomum* Trew
Cinnamon oil^a^	*Cinnamomum cassia*	Branch	Lauraceae	*Cinnamomum* Trew
Clove oil^a^	*Syringa oblata Lindl*	Bud	Oleaceae	*Syringa* Linn.
*Dalbergia* wood oil^a^	*Dalbergia odorifera*	Trunk and root	Leguminosae	*Dalbergia*
*Forsythia suspansa* oil^a^	*Forsythia suspensa*	Fruit	Oleaceae	*Forsythia Vahl*
Holly oil^a^	*Ilex chinensis Sims*	Leaf	Aquifoliaceae	*Ilex* Linn
*Ligusticum wallichi*i oil^a^	*Ligusticum chuanxiong*	Rootstock	Umbelliferae	*Ligusticum* Linn.
*Litsea cubeba* oil^b^	*Litsea cubeba*	Seed	Lauraceae	*Lindera*
Myristica oil^b^	*Myristica ragrans*	Aril	Myristicaceae	*Myristica* Gronov.
Myrrh oil^b^	*Opopanax chrionium*	Resin	Umbelliferae	*Pastinaca* Linn.
Peppermint oil^a^	*Mentha haplocalyx*	Whole plant	Lamiaceae	*Mentha* Linn.
Tea seed oil^a^	*Camellia japonica L*	Fruit	Camelliaceae	*Camellia* Linn.
Thujol oil^c^	*Platycladus orientalis*	Branch and leaf	Cupressaceae	*Platycladus* Spach
Thyme oil^b^	*Thymus vulgaris L*.	Above ground part	Lamiaceae	*Thymus* Linn.

^a,b,c^Means the PEOs were purchased from the Jiangxi Xinsen Natural Vegetable Oil Co. Ltd, Jiangxi Cedar Natural Medicinal Oil Co. Ltd and the Jiangxi Hengcheng Natural Flavor Oil Co. Ltd, respectively.

### Pathogenic fungi

*V*. *virens* (CICC 2710) was bought from the China Center of Industrial Culture Collection (CICC) and cultured on potato dextrose agar medium (PDA, Difco Company) at 4 °C. The pathogen has been identified with PCR by Beijing Majorbio Sanger Bio-pharm Technology Co., Ltd. (See the Supplementary Fig. S3).

### Determination of the fumigation and contact activities of 18 PEOs on mycelial growth

The contact activity of the PEOs was determined using the toxic medium method. First, the stock solution was made by adding 0.04 g PEOs to 10 mL acetone. Second, a 100 μL stock solution of individual PEO was immediately added to the 20 mL sterile PDA medium at 45–50 °C and mixed thoroughly, which was designated toxic medium at the concentration of 20 μg/mL. When the EC_50_ values (the concentration of the tested substance causing a 50% inhibition against the mycelial growth compared with the control) of the PEOs with higher antifungal activity and individual components were determined, appropriate volumes of the stock solution of the PEOs and compounds were adjusted to a series of concentration gradients using acetone. The concentrations tested were 0.250 to 200 μg/mL. The control received the same quantity of acetone mixed with PDA. The 20 mL toxic medium was poured onto three aseptic 6-cm plastic Petri dishes. *V*. *virens* was inoculated immediately by plating a 5 mm diameter disc of the fungus cut with a sterile cork borer from the edge of actively growing cultures on PDA plates in the center of each plate. The Petri dishes were incubated in the dark at 28 °C.

The fumigation activity was determined as described by Soylu *et al*.^40^ with some modifications. Petri dishes (60 × 12 mm, which offer 28 mL air spaces after the addition of 6 mL PDA medium) were used and inoculated with a 5-mm diameter disc of *V*. *virens* in the center. A 0.28 μL aliquot of the PEOs was added to the inner surface of the lid of the Petri dishes, and the final concentration was 10 μL/L air (the EC_50_ values of PEOs with higher antifungal activity and chemicals were determined by pipetting different amounts of the substances tested (0.5 to 6 μL) into the inner surface of the lid of the Petri dishes). The control group was prepared without oil. The plate was immediately sealed with parafilm to prevent any leakage of the PEOs. Each concentration was tested in triplicate. The 5-mm diameter mycelial disc cut from a 15-day-old colony of *V*. *virens* grown on PDA plates and placed upside down in the center of the Petri dishes.

After 7 days of incubation at 28 °C in the dark, the colony growth diameter (mm) was measured using a 150-mm digital caliper. The growth inhibition was calculated using the formula^41^:

$${\rm{Mycelial}}\,{\rm{growth}}\,{\rm{inhibition}}=[({\rm{DC}}-{\rm{DT}})/{\rm{DC}}]\times 100$$

where: DC, DT – average diameter (mm) of the fungal colony of the control and the treatment, respectively. Each test was repeated three times.

### Determination of the fumigation and contact activities of 18 PEOs on conidial germination

The fumigation and contact inhibitory activities of the PEOs on conidial germination were conducted as described by Soylu *et al*.^42^ with slight modification. A spore suspension (10^6^ conidia/mL) of *V*. *virens* was prepared from fermentative potato sucrose broth (PS) as described by Lu *et al*.^43^. A suspension of 80 μL was spread onto the surface of the PDA medium or toxic medium as described in 2.3. The treated concentrations of the PEOs were 7.5 μL/L air for the fumigation activity experiment and 40 μg/mL for the contact activity test. Different concentrations of PEOs with higher antifungal activity (0.5 to 7.5 μL/L air in the fumigation activity test and 32 to 260 μg/mL in the contact activity assay) were applied for the determination of EC_50_ values. The plates were incubated at 28 °C until the conidial germination in the control reached >90% (10–12 h, depending on the rate of germination of *V*. *virens*). Conidial germination was defined as the point at which the germ tube length exceeds the short diameter of the spore. The percentage of conidial germination was estimated under a microscope (Olympus BX51, Tokyo, Japan).

Each test was repeated three times. The percent inhibition was calculated as follows:

$${\rm{Percent}}\,\mathrm{inhibition}\,( \% )=\frac{{\rm{Gc}}-{\rm{Gt}}}{{\rm{Gc}}}\times 100,$$

where Gc and Gt represent the germination rate in control and treated Petri dishes, respectively.

### Analysis of the primary natural components of PEOs with higher antifungal activity

The primary natural components of the strongest PEOs were determined using a gas chromatograph Agilent 7890 A interfaced with a QP2010 5975 C mass spectrometer (Shimadzu). An Rtx-5MS capillary column (30 m × 0.25 mm internal diameter, 0.25 μm film thickness) was used. The column temperature was maintained at 40 °C for 3 min and programmed to 200 °C at a rate of 10 °C/min. The carrier gas was helium with a flow rate of 1 mL/min. The MS were collected at 70 eV and a mass range of 30–550 amu (scan time 0.3 s). Qualitative identification of the compounds of the PEOs was based on retention indices relative to n-alkanes and computer matching with the NIST14. LIB (National Institute of Standards and Technology) in combination with published data. Quantitative analysis of the individual natural major component was based on the peak area normalization measurement, and the relative content of each component was calculated using the % peak area.

### Mycelial morphology observation of *V*. *virens*

Using a clean blade, the square fungal block (5 mm × 5 mm × 2 mm) was cut from the fungal colony incubated in 2.3. The fungal block was dipped into 3.5% glutaraldehyde in 0.1 mol/L phosphate buffer at room temperate for 48 h, and the mycelial block was washed with sterile water four times to rinse off the glutaraldehyde in 30 min. The mycelia were fixed with 1% OsO_4_ for 2 h. The samples were dehydrated in a graded ethanol series (30%, 50%, 70%, 80%, 90%, and 100%) for a period of 20 min in each series and finally treated with 100% ethanol. The samples were dried in a drying apparatus (Leica EM CPD030, Germany) up to the critical point with CO_2_. The fixed material was mounted on stubs using double-sided carbon tape and coated with gold/palladium in a sputter coater system in a high-vacuum chamber (Hitachi MC1000, Japan) for 1 min at 6 mA. The samples were examined, and digital images were captured using an SU 8010 SEM at an accelerating voltage of 12 kV.

### Fungal sporulation difference of *V*. *virens*

The sporulation of *V*. *virens* was tested according to the method described by Sun *et al*.^44^ with some modification. A spore suspension (10^7^ conidia/mL) of *V*. *virens* was prepared from fermentative potato sucrose broth (PS) as described in 4.4. A suspension of 50 μL was spread onto the surface of the PDA medium or toxic medium as described in 2.3. The treated concentrations of the PEOs were 0.5 μL/L air for the fumigation activity experiment and 35 μg/mL for the contact activity test. Plates were sealed with parafilm and incubated at 28 °C and cultured for 10d for sporulation. Fungal spores of each Perti-dish were dislodged with appropriate amount of distilled water and suspended by vigorous mixing for 30 s. The number of spores was determined with a hemacytometer under a microscope (magnification 100×). Each combination of medium and conidial suspension was replicated three times.

### Statistical analysis

The data were analyzed by a one-way analysis of variance (ANOVA) using the SPSS program (IBM SPSS Statistics 22.0). The measurement of the difference was determined using Scheffe’s multiple range test. Values of P < 0.05 were considered statistically significant. Assays were performed in triplicate, and the results were expressed as the mean (±SE). The EC_50_ values were calculated using probit analysis.

## Supplementary information

Supplementary information

## Data Availability

Te datasets generated and analysed during the current study are not publicly available due to possible valorisation of the data but are available from the corresponding author on reasonable request.
