# Supplementary material for: Fumigation and contact activities of 18 plant essential oils on Villosiclava virens, the pathogenic fungus of rice false smut
Source: Sci Rep. 2019 May 14;9:7330. doi: 10.1038/s41598-019-43433-x (PMC6517416; doi:10.1038/s41598-019-43433-x)
Supplement: Supplementary file 1 — Supplementary information [file 41598_2019_43433_MOESM1_ESM.pdf]

Supplementary information

Fumigation and contact activities of 18 plant essential oils on *Villosiclava virens*, the pathogenic fungus of rice false smut

Jingge Zheng, Tingting Liu, Zhixin Guo, Lan Zhang, Liangang Mao, Yanning Zhang, Hongyun Jiang*

Key Laboratory of Control of Biological Hazard Factors (Plant Origin) for Agri-product Quality and Safety, Ministry of Agriculture and Rural Affairs of the People's Republic of China, Institute of Plant protection, Chinese Academy of Agricultural Sciences, Beijing, 100193, China

Correspondence and requests for materials should be addressed to H.J. (Phone: +8610-62893622; E-mail: ptnpc@vip.163.com

This supplementary information file contains one supplemental table and three supplemental figures.

Figure S1 The photographs with light microscope of *Villosiclava virens* hyphae. The pictures on the top are treated at 0.5 μL/L air in the fumigation activity assay. A, Hyphae without PEOs or cinnamaldehyde (control). B-D, Hyphae treated with cinnamon bark oil, cinnamon oil and trans-cinnamaldehyde. On the bottom are treatments from the contact activity experiment at a concentration of 15 μg/mL. a, Hyphae treated with acetone (CK). b-d, Hyphae treated with cinnamon bark oil, cinnamon oil and *trans*-cinnamaldehyde. All of the magnifications are ×400.

Figure S2 The photographs with light microscope of *Villosiclava virens* conidial spores. The pictures on the top are treated at 0.5 μL/L air in the fumigation activity assay. A, Conidial spores without PEOs or cinnamaldehyde (control). B-D, Conidial spores treated with cinnamon bark oil, cinnamon oil and *trans*-cinnamaldehyde. On the bottom are treatments from the contact activity experiment at a concentration of 35 μg/mL. a, Conidial spores treated with acetone (CK). b-d, Conidial spores treated with cinnamon bark oil, cinnamon oil and *trans*-cinnamaldehyde. All of the magnifications are ×100.

Figure S3 The results of identification specific gene sequences of the pathogen by agarose gel electrophoresis. The arrow pointed at the ribbon of the pathogen named as U.V. M means DL2,000 DNA marker.

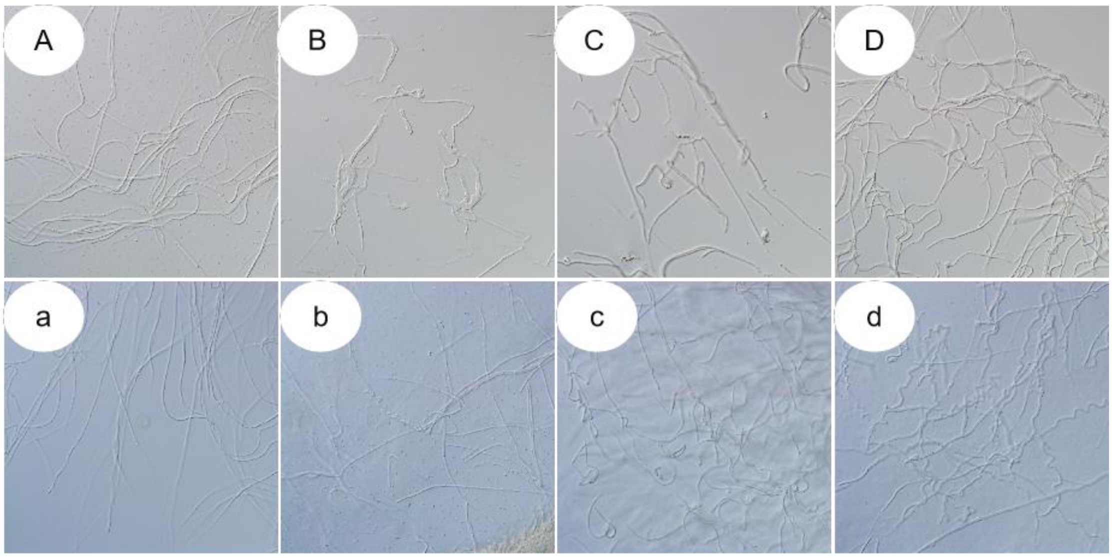
A
B
C
D
a
b
c
d

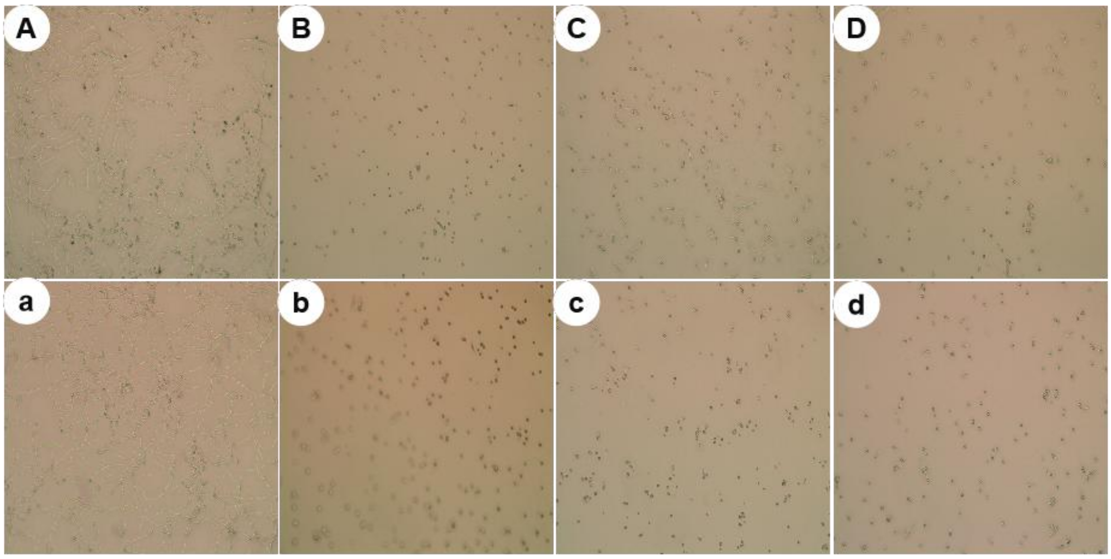
A
B
C
D
a
b
c
d

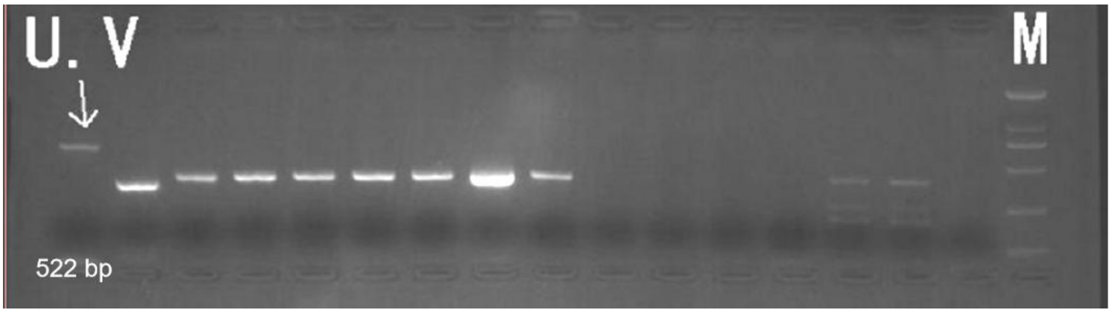
U. V
M
522 bp

Table S1 The detailed data of conidial germination and inhibition in different treatment.(Na = the number of germinated conidia in the treated Petri plate, Nb = the number of all the germinated conidia in the treated Petri plate).

**Concentration of essential oils (μg/mL)**	**Germination rate (= 100* Na/Nb)**			**Average germination (%)**	**Inhibition (%)**
Control	100	100	100	100	0.00
Cinnamon oil - 28	82.6	88.4	64.0	78.3	21.7
Cinnamon oil - 30	57.0	73.0	69.0	66.3	33.7
Cinnamon oil - 32	49.0	29.0	53.0	43.7	56.3
Cinnamon oil - 34	11.0	13.0	25.0	16.3	83.7
Cinnamon oil - 36	3.00	1.00	1.00	1.67	98.3
Cinnamon bark oil - 32	86.9	64.4	44.3	65.1	34.9
Cinnamon bark oil - 34	52.4	50.8	28.5	43.9	56.1
Cinnnamon bark oil-36	5.80	4.00	5.00	5.00	95.0
Cinnamon bark oil -38	7.90	4.00	1.00	4.50	95.5
Trans-cinnamaldehyde-32	98.1	99.2	95.4	97.5	2..50
Trans-cinnamaldehyde-34	91.9	93.7	94.3	93.3	6.70
Trans-cinnamaldehyde-36	17.4	42.7	45.5	35.2	64.8
Trans-cinnamaldehyde-38	21.2	18.3	19.7	19.7	80.3
